# Supplementary material for: Single-cell glycomics analysis by CyTOF-Lec reveals glycan features defining cells differentially susceptible to HIV
Source: eLife. 2022 Jul 5;11:e78870. doi: 10.7554/eLife.78870 (PMC9255966; doi:10.7554/eLife.78870)
Supplement: Supplementary file 1. [file elife-78870-supp1.docx]

**SUPPLEMENTARY FILE 1 (SUPPLEMENTARY TABLES)**

**Supplementary File 1A. List of CyTOF antibodies**

| **Antibody** | **Metal label** | **Clone** | **Vendor** |
| --- | --- | --- | --- |
| AOL* | 141Pr | N/A | In-house |
| CD19 | 142Nd | HIB19 | Fluidigm |
| CCR5 | 144Nd | NP6G4 | Fluidigm |
| CD8 | 146Nd | RPAT8 | Fluidigm |
| CD7 | 147Sm | CD76B7 | Fluidigm |
| ICOS | 148Nd | C398.4A | Fluidigm |
| HSA | 150Nd | M1/69 | In-house |
| MAL-1* | 151Eu | N/A | In-house |
| WGA* | 152Sm | N/A | In-house |
| CD62L | 153Eu | DREG56 | Fluidigm |
| TIGIT | 154Sm | MBSA43 | Fluidigm |
| CCR6 | 155Gd | G034E3 | In-house |
| UEA-1* | 156Gd | N/A | In-house |
| OX40 | 158Gd | ACT35 | Fluidigm |
| CCR7 | 159Tb | G043H7 | Fluidigm |
| CD28 | 160Gd | CD28.2 | Fluidigm |
| CD45RO | 161Dy | UCHL1 | In-house |
| CD69 | 162Dy | FN50 | Fluidigm |
| CRTH2 | 163Dy | BM16 | Fluidigm |
| PD1 | 164Dy | EH12.1 | In-house |
| CD127 | 165Ho | A019D5 | Fluidigm |
| CXCR5 | 166Er | RF8B2 | In-house |
| CD27 | 167Er | L128 | Fluidigm |
| CD30 | 168Er | BerH8 | In-house |
| CD45RA | 169Tm | HI100 | Fluidigm |
| CD3 | 170Er | UCHT1 | Fluidigm |
| ABA* | 171Yb | N/A | In-house |
| CD38 | 172Yb | HIT2 | Fluidigm |
| α4β7 | 173Yb | Act1 | In-house |
| CD4 | 174Yb | SK3 | Fluidigm |
| CXCR4 | 175Lu | 12G5 | Fluidigm |
| CD25 | 176Yb | M-A251 | In-house |
| CLA | 209Bi | HECA-452 | In-house |
| HLADR | 112Cd | Tu36 | Invitrogen |
| RORγt^#^ | 115Di | AFKJS-9 | In-house |
| NFAT1^#^ | 143Nd | D43B1 | Fluidigm |
| BIRC5^#^ | 145Nd | 91630 | In-house |
| Tbet^#^ | 149Sm | eBio4B10 (4B10) | In-house |
| CTLA4^#^ | 157Gd | 14D3 | In-house |

**lectins,* *^#^Intracellular antibodies*

**Supplementary File 1B. Binding properties of lectins used in this study**

| **Property** | **MAL-1** | **WGA** | **UEA-1** | **AOL** | **ABA** |
| --- | --- | --- | --- | --- | --- |
| Full Name/Source | Maackia Amuren-sis I | Wheat Germ Agglutinin | Ulex Euro-paeus I | Aspergillus Oryzae | Agaricus Bisporus Agglutinin |
| Subunits Number | 2 | 2 | 2 | 1 | 4 |
| Molecular Weight (Da) | 130 k | 36 k | 63 k | 35 k | 68 k |
| Carbohydrate specificity | Sia(α2-3)Gal (β1-4) GlcNAc | Sialic acid/ β1-4  GlcNAc | α1-2 branched fucose | total/α1-6 core fucose | T antigen, Galβ1-3GalNAcα1 |
| Sialic acids binding | α2-3 linked | Total | No | No | No |

*GlcNAc: N-acetylglucosamine, GalNAc: N-acetylgalactosamine*
